# Urinary brain-derived neurotrophic factor and nerve growth factor as noninvasive biomarkers of overactive bladder in children

**DOI:** 10.11613/BM.2022.030706

**Published:** 2022-10-01

**Authors:** Merima Colic, Dunja Rogic, Jasna Lenicek Krleza, Ana Kozmar, Lorna Stemberger Maric, Slaven Abdovic

**Affiliations:** 1 Department of Pediatric Nephrology, Children’s Hospital Zagreb, Zagreb, Croatia; 2 Department of Laboratory Diagnostics, University Hospital Center Zagreb, Zagreb, Croatia; 3 Department of Laboratory Diagnostics, Children’s Hospital Zagreb, Zagreb, Croatia; 4 Pediatric Infectious Diseases Department, University Hospital for Infectious Diseases “dr. Fran Mihaljevic”, Zagreb, Croatia

**Keywords:** biomarker, brain-derived neurotrophic factor, child, nerve growth factor, overactive bladder

## Abstract

**Introduction:**

Overactive bladder (OAB) is the most common urinary disorder and the leading cause of functional daytime intermittent urinary incontinence in children. The aim of this study was to determine whether urinary brain-derived neurotrophic factor (BDNF) and nerve growth factor (NGF) concentrations, normalized to urine creatinine, could be used as biomarkers for diagnosis and treatment monitoring of OAB in children.

**Materials and methods:**

Urine samples of 48 pediatric patients with OAB were collected at the start of anticholinergic therapy (baseline), at follow-up visits (3 and 6 months), and from 48 healthy controls. Urinary BDNF and NGF concentrations were determined by ELISA method (Merck, Darmstadt, Germany) and Luminex method (Thermo Fisher Scientific, Waltham, USA). Differences of frequency between quantifiable analyte concentrations between subject groups were determined using Fisher’s exact test.

**Results:**

There was no statistically significant difference between quantifiable analyte concentrations between patients at baseline and the control group for BDNF and NGF by either the ELISA or Luminex method (P = 1.000, P = 0.170, P = 1.000, and P = N/A, respectively). There was a statistically significant difference between quantifiable BDNF by the ELISA method between patients at baseline and complete success follow-up (P = 0.027), while BDNF by Luminex method and NGF by both methods were not statistically significant (P = 0.078, P = 0.519, and P = N/A, respectively).

**Conclusions:**

This study did not demonstrate that urinary BDNF and NGF concentrations, can be used as biomarkers for diagnosis and therapy monitoring of OAB in children.

## Introduction

Overactive bladder (OAB) is the most common urinary tract disorder and the leading cause of functional daytime intermittent urinary incontinence, with a prevalence of 9% in children aged 5-14 years (*1*). The obligatory symptom is urgency, which may be accompanied by frequent urination during the day, daytime intermittent urinary incontinence, enuresis, and nocturia in the absence of a urinary tract infection (*2*). In most cases, the diagnosis of OAB is based solely on clinical symptoms, mainly because of the invasiveness of urodynamic studies which may cause pain, discomfort, and lack of cooperation from the child. In addition, detrusor overactivity (DO) is a pathognomonic finding during urodynamics, but it is present in only 50-70% of affected individuals (*3*, *4*). Considering the high prevalence, the negative impact on the quality of life of the child and their family, the economic burden, and the fact that OAB in childhood is a strong predisposing factor for OAB in adulthood, it is important to improve the objective and noninvasive diagnosis and treatment monitoring of OAB in children (*5*-*7*).

The pathogenesis of OAB is not fully understood, but studies from the last decade have indicated the role of neurotrophins in the mechanisms of manifestation of this lower urinary tract dysfunction (*8*). Neurotrophins belong to a large family of trophic factors that are critical for the survival of neurons and the formation, maintenance, and regulation of synapses. The most studied representatives of neurotrophins in lower urinary tract disorders are nerve growth factor (NGF) and brain-derived neurotrophic factor (BDNF). In several clinical studies of adult patients with OAB, a statistically significant higher concentration of NGF was found in the urine of patients compared with healthy controls (*9*). Urinary NGF concentration correlated proportionally with symptom severity (*10*, *11*). After successful anticholinergic treatment, urinary NGF concentrations were significantly lower in both adults and children (*3*, *10*, *12*-*15*). Nevertheless, high concentrations of urinary NGF persisted in patients with poor response to therapy, probably because of the presence of chronic inflammation of the bladder wall (*16*). In these studies, the enzyme-linked immunosorbent assay (ELISA) method was used to detect neurotrophins.

Despite multiple studies showing that patients with OAB have significantly higher urinary concentrations of NGF and BDNF, several recent studies have yielded conflicting results. On the one hand, some studies found no significant difference in the NGF/creatinine ratio between patients with OAB and the control group (*17*, *18*). On the other hand, other studies showed unmeasurable NGF concentrations using various commercially available ELISA kits (*19*-*21*). These studies have drawn attention to the importance of using multiple methods to establish optimal procedures for biomarker detection in urine. A 2019 meta-analysis on the use of urinary NGF as a biomarker for DO in children notes that it is a potential noninvasive marker but highlights the need for further clinical studies to confirm this hypothesis (*22*).

The aim of this study was to determine whether BDNF and NGF concentrations, normalized to urine creatinine, could be used as diagnostic and treatment-monitoring biomarkers for OAB in the pediatric population.

The first hypothesis was that urinary BDNF and NGF concentrations normalized to urine creatinine would be higher in children with OAB before anticholinergic therapy than in the control group. The second hypothesis was that urinary BDNF and NGF concentrations normalized to urinary creatinine would be higher in children with OAB before anticholinergic treatment than after successful treatment.

## Materials and methods

### Subjects

This was a prospective intervention study that included 48 outpatients with urgency aged 4-17 years who were consecutively examined between February 2020 and November 2021. The urgency was defined as a sudden and unexpected experience of an immediate and compelling need to urinate after achieving bladder control and had to be noted more than once *per* week for at least 3 months. Exclusion criteria were acute urinary tract infection (UTI), central or peripheral nervous system disorders, lumbosacral anomalies, surgical procedures, or urinary or genital tract anomalies, diabetes mellitus, diabetes insipidus, polydipsia (> 2 L/m^2^/24 h), and anticholinergic treatment in the previous 3 months.

The control group consisted of 48 inpatients recruited from different pediatric departments between June 2019 and November 2020. They had normal micturition behavior and clinical examination with no symptoms or confirmed lower urinary tract disease, no history of urinary tract infection, chronic disease, or urinary tract or genital abnormalities.

The protocol for the research project was approved by the Institutional Ethics Committee (Approval No. 02-26/10-8-1), and it conforms to the provisions of the Declaration of Helsinki. Informed consent was obtained from the participants and their legal guardians before the study procedures. The study was registered at ClinicalTrials.gov under NCT02704013 (ID: pOAB-CHZ-01).

### Methods

The screening examination for OAB patients included a history, physical examination, 2-day frequency-volume chart (FVC), ultrasound examination of the kidneys and bladder, uroflow examination with determination of residual urine volume after urination (*post*-void residual, PVR), urinalysis, and urine culture. Prior to the start of the study, participants with constipation were treated with osmotic laxatives, behavioral modifications, and dietary changes. All patients received standard urotherapy which includes the following components:

1. Information about the normal function of the lower urinary tract (LUT) and how the child in question deviates from the norm,
2. Behavior modification instructions such as regular voiding habits, correct voiding posture, avoidance of holding maneuvers, regular bladder and bowel emptying patterns,
3. Lifestyle advice such as balanced fluid intake and diet, reduced caffeine intake,
4. Recording of symptoms and voiding habits in bladder diaries or frequency-volume charts, and
5. Support and encouragement through regular follow-up with the caregiver (*23*).

Urodynamics was recommended to all patients who had persistent urinary urgency at baseline. Urodynamics was performed according to International Children’s Continence Society (ICCS) guidelines and good practice (*23*). All enrolled patients received oral oxybutynin (0.3-0.5 mg/kg/day bid) or solifenacin (5 mg oral once daily for children aged ≥ 6 years) as initial therapy. Standard urotherapy was continued in all patients during drug treatment. Follow-up examinations were recommended at month 3 and month 6, during which the treatment outcomes were assessed using the ICCS classification (*23*). Patients in whom symptoms remitted 100% were defined as a complete success, patients in whom symptoms remitted 50-99% were defined as a partial success, and patients in whom symptoms remitted less than 50% were defined as nonresponders.

The urine samples of the subjects and the control subjects were taken at the subjective sensation of a full bladder and the urge to void. The samples were midstream clean catch samples collected in sterile urine collection cups with screw caps at room temperature. One millilitre of the sample was used for the analysis of creatinine concentration in urine on the Olympus AU680 automated biochemistry analyser (Beckman Coulter, Brea, USA) by the enzymatic method within one hour of collection. Ten millilitres of urine was centrifuged at 3000xg for 10 minutes at room temperature within 30 minutes of collection. Five 1.5 mL aliquots of the supernatant were frozen and stored at - 80 °C until analysis of neurotrophin concentrations. The aliquots were thawed only once; there were no cases of multiple freeze-thaw cycles.

The concentrations of BDNF and NGF were analysed by the ELISA method (Merck, Darmstadt, Germany) and Luminex method (Thermo Fisher Scientific, Waltham, USA) according to the manufacturers’ instructions.

Enzyme-linked immunosorbent assay is an *in vitro* enzyme-linked immunoassay for the quantitative measurement of a target protein in biological samples. In this study, the ELISA 96 well plate is coated with specific capture antibodies (against BDNF or NGF). Standard solutions and samples are pipetted into the wells and the target protein present in a sample is bound to the wells by the immobilized antibody. The wells are washed and a biotinylated detection antibody specific for the target protein is added. After washing out the unbound biotinylated antibody, horseradish peroxidase (HRP)-conjugated streptavidin is pipetted into the wells. The wells are washed again, a 3,3′,5,5′-tetramethylbenzidine (TMB) substrate solution is added to the wells, and the color develops in proportion to the amount of bound target protein. The stop solution changes color from blue to yellow, and the absorbance in each well is measured by a microplate reader at 450 nm. The concentration of the target protein in the samples is calculated from the standard curve generated from the known concentrations and the absorbance obtained from the standard solutions used. The detection range of the ELISA kits used for this study was 0.07-16.00 ng/mL for BDNF, while the detection range for NGF was 6.86-5000.00 pg/mL. Quality control was not performed during the analyte investigation. The analyses were performed within two years of sample collection. Protein stability was not investigated prior to analysis. BDNF and NGF concentrations were analysed by the ELISA methods concurrently from the same aliquot.

The Luminex method is a multiplex sandwich bead-based immunoassay that combines the ELISA method with flow cytometry. It uses magnetic beads that are internally dyed with a graded mixture of fluorescent dyes. Varying the level of internal staining of the beads produces hundreds of different fluorescence profiles that can be individually interrogated and classified in a single sample. Microspheres of a single identity are conjugated on their surface to a specific capture antibody for a protein of interest. A mixture of beads is pipetted into a 96-well plate. After incubation of the sample and beads, the wells are washed and a cocktail of biotinylated detection antibodies specific for the target proteins is added. After washing out the unbound biotinylated antibodies, phycoerythrin (PE)-conjugated streptavidin is pipetted into the wells. After another round of washing, the microsphere-cytokine reporter compound is analysed as a single sphere suspension through a flow chamber equipped with excitation lasers and an electronic detection device that measures the intensity of fluorescence of the microsphere-cytokine reporter compound, similar to a flow cytometer. Proteins are distinguished by the color of the internally stained beads themselves, and their relative abundance is quantified by measuring the fluorescence intensity of the detection antibody conjugated to the reporter dye. The detection range of the Luminex kit used for this study was 1.33-5450 pg/mL for BDNF, while the detection range for NGF was 7.35-30,100 pg/mL. Quality control was not performed during the analyte investigation. The analyses were performed within two years of sample collection. Protein stability was not investigated prior to analysis. BDNF and NGF concentrations were analysed with the same Luminex assay.

### Statistical analysis

Because analyte concentrations were unquantifiable in the majority of samples, the neurotrophin concentration results were converted to categorical variables. Concentrations of BDNF were determined by the ELISA method and were converted into categories of < 0.07 ng/mL and ≥ 0.07 ng/mL. Also, BDNF concentrations determined by the Luminex method were converted into categories of < 1.33 pg/mL and ≥ 1.33 pg/mL. Concentrations of NGF were determined by the ELISA method and were converted into categories of < 6.86 pg/mL and ≥ 6.86 pg/mL. Also, NGF concentrations determined by the Luminex method were converted into categories of < 7.35 pg/mL and ≥ 7.35 pg/mL. Descriptive statistics were used to describe the demographic and disease data of the study participants. The age of the subjects was expressed as median and range (min-max). Frequencies of sex in the patient and control group, quantifiable neurotrophin concentration results, and complete success rate were expressed as percentages for sample sizes > 100, ratios for sample sizes > 30 and < 100, and as the number of the observations divided with the total number of subjects within the group. The differences between the concentrations of BDNF and NGF measured by both methods were compared between the baseline group of patients and the control group was determined to test the first hypothesis. The differences between the concentrations of BDNF and NGF measured by both methods between the baseline group of patients and the follow-up group of patients with complete success was determined to test the second hypothesis. Differences of frequency between categorical variables (*i.e.,* analyte concentrations) between subject groups were determined using Fisher’s exact test. Statistical analysis was performed with the statistical program MedCalc, version 18.9 (MedCalc Software, Ostend, Belgium). P values < 0.05 were considered statistically significant.

## Results

The median age of the patient group was 7 (3-17) years. The median age of the control group was 13 (6-17) years. The patient group consisted of 43 (0.90) girls and 5 (0.10) boys. The control group consisted of 19 (0.40) girls and 29 (0.60) boys.

Of the 48 patients recruited, 35 patients completed their follow-up at month 3 and 33 patients completed their follow-up at month 6. At follow-up visits, 18 (0.38) patients had complete initial success either 3 or 6 months since the start of anticholinergic therapy. This is in accordance with previously published data on the success of anticholinergic treatment of children with OAB (*24*).

Of the 164 samples analysed, a majority of samples had unquantifiable analyte concentrations regardless of a subject group (patients with OAB or controls) or method used (ELISA or Luminex). These concentrations are considered to be less than the lowest analysed concentration standard. Overall, the concentration of BDNF in urine was < 0.07 ng/mL in 150 (91%) of the samples analysed by the ELISA method, while the Luminex method showed a BDNF concentration of < 1.33 pg/mL in 121 (74%) samples. Urinary NGF concentration was < 6.86 pg/mL in 158 (96%) samples analysed by the ELISA method, while all 164 (100%) samples had a concentration of < 7.35 pg/mL by the Luminex method.

Table 1 shows the overview of the number of quantifiable BDNF and NGF concentrations across subject groups using the ELISA and Luminex methods.

**Table 1:** Overview of the number of quantifiable BDNF and NGF concentrations across healthy controls and patient groups using the ELISA and Luminex methods

**Analyte and method**	**Category**	**Patients baseline** **(N = 48)**	**Patients** **follow-up - nonresponders and partial success** **(N = 47)**	**Patients** **follow-up -** **complete success** **(N = 21)**	**Control group** **(N = 48)**
BDNF	≥ 0.07 ng/mL, N	1 (0.02)	8 (0.17)	4/21	1 (0.02)
ELISA	< 0.07 ng/mL, N	47 (0.98)	39 (0.83)	17/21	47 (0.98)
BDNF	≥ 1.33 pg/mL, N	5 (0.10)	21 (0.45)	6/21	11 (0.23)
Luminex	< 1.33 pg/mL, N	43 (0.90)	26 (0.55)	15/21	37 (0.77)
NGF	≥ 6.86 pg/mL, N	1 (0.02)	4 (0.09)	1/21	0 (0.00)
ELISA	< 6.86 pg/mL, N	47 (0.98)	43 (0.91)	20/21	48 (1.00)
NGF	≥ 7.35 pg/mL, N	0	0	0/21	0
Luminex	< 7.35 pg/mL, N	48 (1.00)	47 (1.00)	21/21	48 (1.00)
N – number. BDNF – brain-derived neurotrophic factor. NGF – nerve growth factor. ELISA – enzyme-linked immunosorbent assay.					

Table 2 shows the results of Fisher’s exact test between subject groups, analytes, and methods.

**Table 2:** Results of Fisher’s exact test

**Analyte**	**Method**	**Patients baseline *vs*. control group**	**Patients baseline *vs*. patients** **follow-up - complete success**
BDNF	ELISA	P = 1.000	P = 0.027
	Luminex	P = 0.170	P = 0.078
NGF	ELISA	P = 1.000	P = 0.519
	Luminex	NA	NA
P values were calculated using Fisher’s exact test. BDNF – brain-derived neurotrophic factor. NGF – nerve growth factor. ELISA – enzyme-linked immunosorbent assay. NA – not applicable.			

The only statistically significant difference in frequency of quantifiable concentrations of BDNF using the ELISA method was found between the patient group baseline and patients with complete success at follow-up (P = 0.027).

An additional analysis of individual patient assessments showed that of the 18 patients with complete success at follow-up, four patients had quantifiable BDNF concentration with at follow-up but not baseline.

## Discussion

In contrast to most previously published studies, this study did not find a statistically significant difference between urinary BDNF and NGF concentrations in OAB patients before treatment and control subjects. Consequently, it does not support their use as noninvasive diagnostic biomarkers for OAB.

There was no statistically significant difference between patients’ NGF concentrations before therapy and at complete success follow-up, so this study does not support its use as a biomarker to monitor therapy in children with OAB.

Because a statistically significant difference in the frequency of BDNF concentrations quantifiable by the ELISA method was found between the group of patients at baseline and those with complete success at follow-up (P = 0.027), additional analysis of individual patient assessments was performed. In contrast to the expected results of a decrease in BDNF concentration after successful anticholinergic therapy, no patient with complete success showed a decrease in concentration at follow-up. As indicated in the results, four patients had quantifiable BDNF concentration at follow-up but not at baseline. Therefore, this study could not confirm the usefulness of urinary BDNF as a biomarker for monitoring therapy in children with OAB because it was not possible to determine the decline in concentrations in successfully treated patients.

These results are not consistent with those of most previously published studies. There are several factors that should be considered when interpreting this difference. One of the most important is the limited sensitivity of current ELISA technology for determining urinary BDNF and NGF concentrations. In their letter to the editor on a meta-analysis on the usefulness of NGF as a biomarker in urine OBA, Gamper and Viereck emphasise that many studies used ELISA kits from Promega (Promega, Madison, USA) that were later shown to quantify urine components other than NGF and were therefore withdrawn from the market (*25*). They also refer to two of the above-mentioned studies in which NGF concentrations were below the limit of detection, which is consistent with our study (*20*, *21*).

Given that the Luminex technology has similar detection limits for NGF concentrations, it is not surprising that it gives similar results. On the other hand, the detection limit for BDNF concentrations is lower, so it was possible to quantify it in more samples. However, in our study, only 43 (26%) of the samples had quantifiable BDNF concentrations using this method, so we conclude it is not yet sensitive enough to use BDNF as a biomarker for OAB in children.

Another factor to consider is the preanalytical process from sample collection to neurotrophin analysis. One of the possible reasons for low concentrations could be that proteins are digested by proteases before centrifugation and freezing at - 80°. Previously published studies showing quantifiable concentrations of neurotrophins did not report the use of protease inhibitors, so we did not include their use in our study protocol. Another preanalytical element that may affect the detection of proteins is the time from sample collection to storage and analysis. In our study protocol, the maximum time from sample collection to centrifugation and storage at - 80 °C was 30 min. Although shortening the time could be further tested, we believe that a shorter time, such as 15 minutes, is not feasible for routine laboratory testing, should these proteins be used regularly as biomarkers for OAB. An additional element in the study protocols that could be considered is the temperature during centrifugation. We centrifuged samples at room temperature, as did some of the cited studies, whereas other investigators centrifuged them at 4 °C (*3*, *10*-*13*, *16*, *17*, *20*, *21*). Since both protocols yielded both measurable and unquantifiable concentrations of neurotrophins, we do not believe that the temperature of centrifugation affected the results of the analysis of urinary BDNF and NGF concentrations (*3*, *10*-*13*, *16*, *17*, *20*, *21*).

A limitation of this study was that neurotrophin concentrations were analysed at different time intervals after sample collection. To our knowledge, there are no studies on the stability of neurotrophins in urine samples stored at - 80 °C. It was unfeasible to standardise the interval from collection to analysis because this was a prospective intervention study in the paediatric population and the target proteins were analysed with immunoassays performed on batches of 80 samples. A second limitation was that the patient and control groups were not matched for age and sex. The reason for this limitation was that this study was performed as part of a larger study funded by the Croatian Science Foundation, in which one of the objectives was to determine the age and sex variations of neurotrophins in healthy children. Therefore, it was not possible to have a disproportionate number of girls in the control group as in the patient group.

Taking into consideration the time and resources that have been devoted to researching the role of neurotrophins in the pathology of OAB, the question is what to do next. One possibility is to develop immunochemical assays with a lower limit of detection that are more suitable for urine analyses. Another option is to use more sensitive methods such as tandem mass spectrometry. However, since the ultimate goal in determining new urinary biomarkers for diagnosis and treatment monitoring is that they be widely available and inexpensive, methods that are difficult to use and expensive are not of interest. Alternatively, the research community may turn its attention to novel target proteins such as inflammatory cytokines and chemokines in urine. Their study could help us determine the pathophysiology of OAB and the role that inflammation plays in it. One of the potential biomarkers studied in recent years is monocyte chemoattractant protein-1 (MCP-1), a chemokine that regulates monocyte/macrophage migration and infiltration. Some studies showed increased MCP-1 concentrations in the urine of adult patients with OAB compared to the control group (*26*, *27*). To our knowledge, there has been no study of inflammatory cytokines and chemokines in the urine of children with OAB.

Our study does not support the hypothesis that urinary BDNF and NGF concentrations, normalized to urine creatinine, are higher in children with OAB before anticholinergic therapy than in the control group. In addition, the hypothesis that urinary BDNF and NGF concentrations normalized to urine creatinine are higher in children with OAB before anticholinergic treatment than after successful treatment could not be confirmed.

## Data Availability

The data generated and analysed in the presented study are available from the corresponding author on request.
